# Chronic Drinking During Adolescence Predisposes the Adult Rat for Continued Heavy Drinking: Neurotrophin and Behavioral Adaptation after Long-Term, Continuous Ethanol Exposure

**DOI:** 10.1371/journal.pone.0149987

**Published:** 2016-03-01

**Authors:** Gina M. Fernandez, William N. Stewart, Lisa M. Savage

**Affiliations:** Department of Psychology, Binghamton University, State University of New York, Binghamton, New York, United States of America; Radboud University Medical Centre, NETHERLANDS

## Abstract

Previous research has found that adolescent ethanol (EtOH) exposure alters drug seeking behaviors, cognition and neuroplasticity. Using male Sprague Dawley rats, differences in spatial working memory, non-spatial discrimination learning and behavioral flexibility were explored as a function of age at the onset (mid-adolescent vs. adult) of chronic EtOH exposure (CET). Concentrations of mature brain-derived neurotrophic factor (mBDNF) and beta-nerve growth factor (β-NGF) in the prefrontal cortex and hippocampus were also assessed at different time-points: during CET, following acute abstinence (48-hrs), and after protracted abstinence (6–8 wks). Our results revealed that an adolescent onset of CET leads to increased EtOH consumption that persisted into adulthood. In both adult and adolescent onset CET groups, there were significant long-term reductions in prefrontal cortical mBDNF and β-NGF levels. However, only adult onset CET rats displayed decreased hippocampal BDNF levels. Spatial memory, assessed by spontaneous alternation and delayed alternation, was not significantly affected by CET as a function of age of drinking onset, but higher blood–EtOH levels were correlated with lower spontaneous alternation scores. Regardless of the age of onset, EtOH exposed rats were impaired on non-spatial discrimination learning and displayed inflexible behavioral patterns upon reversal learning. Our results indicate that adolescent EtOH exposure changes long-term consumption patterns producing behavioral and neural dysfunctions that persist across the lifespan.

## Introduction

Approximately 17 million adults suffer from an alcohol use disorder (AUD) and the majority of adults diagnosed with an AUD began consuming alcohol during adolescence [1]. Adolescence is identified as a vulnerable developmental time period during which exposure to drugs, including alcohol, can have long lasting effects on memory, cognition, anxiety and social interaction [2–4]. Early ethanol (EtOH) exposure appears to solidify adolescent-typical behaviors in rodents, such as increased impulsivity, decreased behavioral inhibition, and behavioral inflexibility, well into adulthood [5,6]. These unique behavioral effects are sustained through long-lasting neural changes in critical brain regions such as the frontal cortex and the hippocampus [7,8]. Thus, early EtOH exposure can produce a state wherein the adult brain is primed for later chronic alcohol abuse and associated behavioral impairment [9].

The hippocampus is vulnerable to chronic EtOH exposure and these alterations contribute to memory deficits associated with alcoholism [10–13]. In rodents, chronic exposure to EtOH results in reduced interneuron, pyramidal and granule cell numbers [14–16], as well as reduced long-term potentiation within in the hippocampus [17,18]. Hippocampal neurogenesis is also very sensitive to chronic EtOH exposure. Several chronic EtOH delivery paradigms, both intermittent and continuous, decrease the number of newly born surviving neurons (by 40–60%) within the dentate gyrus [12,19]. High blood ethanol concentrations (BECs) are needed to observe reductions in neurogenesis [20]. Because newborn hippocampal neurons in adolescent rats are less likely to incorporate into a functional neural network following excessive EtOH treatment, the adolescent hippocampus displays a distinct vulnerabililty to chronic EtOH [21]. In human and non-human primates, hippocampal volume loss is greater following adolescent-onset alcohol consumption, compared with adult-onset [22,23]. These EtOH induced changes in hippocampal structure are believed to contribute to the initial spatial processing, episodic learning, and memory impairments observed in alcoholics [24].

The frontal cortex is also adversely affected by EtOH exposure, perhaps to a greater degree than the hippocampus [25–27]; a region that is developmentally sensitive to EtOH toxicity [28]. The extent of reduction in frontal cortical volume is largely dependent upon the extent of alcohol exposure [28–31]. Shrinkage of the frontal cortex in alcoholics is related to reduced neuronal size, branching of basal dendrites [32] and glial cell density [33]. Markers of cell death are increased in the prefrontal cortex of alcoholics, suggesting alcohol-related neural degeneration within this region [34]. Chronic EtOH exposure in adolescence leads to the induction of a persistent neural immune response within the frontal cortex that contributes to alcohol-related brain damage [35]. In both humans and nonhuman mammals, increased EtOH consumption correlates with decreases in behavioral flexibility and response inhibition, behaviors that are modulated by the frontal cortex [36–39].

EtOH is known to disrupt several signaling cascades and one target effector is BDNF [40]. Abnormalities in neurotrophins are associated with cognitive deterioration [41,42] and neuronal atrophy [43]. In particular, brain-derived neurotrophic factor (BDNF) and nerve growth factor (NGF) are of interest due to their role in homeostatic neural function and recovery during and after EtOH exposure [44–47]. Previous research indicates a biphasic temporal effect of EtOH on BDNF and NGF levels: short exposures appear to increase levels, while prolonged exposures seem to reduce levels [44]. In addition, BDNF levels are not changed during excessive adolescent binge EtOH exposure [21]. Altered levels of neurotrophins appear to contribute to alcohol-related brain damage and affiliated cognitive impairment [48].

Given the potential interactions between the timing of EtOH exposure, brain regional sensitivity, and neurotrophin levels, the current project investigated whether age differences (mid-adolescence versus adulthood) at onset of CET would modulate the degree of neural and behavioral dysfunction typically associated with AUDs. Chronic EtOH exposure in drinking water has been used as a model to evaluate the effects of long-term alcohol abuse observed in middle-aged to advanced-aged populations [49,50]. Specifically, this model was instrumental in revealing that the forebrain cholinergic population and cholinergic innervation of the hippocampus and cortex are very sensitive to long-term EtOH toxicity. CET models have also demonstrated that exogenous NGF application improved neural and behavioral outcomes after CET [51–55].

We monitored total EtOH consumption throughout a 6-month forced EtOH exposure paradigm, followed by a battery of behavioral tests that included spatial and non-spatial discrimination learning, as well as reversal learning as a measure of behavioral flexibility. Visuospatial function has been reported to be initially impaired in abstinent alcoholics and is associated with reduced hippocampal volume [24]. Behavioral or cognitive flexibility, which is dependent on the frontal cortex, is also impaired in abstinent alcoholics [56]. Thus, tasks were chosen that are dependent on hippocampal and frontal cortical functioning. Neurotrophic factors (mBDNF, β-NGF) were measured within the frontal cortex and hippocampus at three time-points to examine neural adaptions as a function of intoxication, withdrawal and protracted abstinence. It was hypothesized that initiation of chronic EtOH exposure during adolescence would produce persistent disruptions in drinking patterns, neurotrophin levels and cognitive function.

## Materials and Methods

### Ethics Statement, Minimization of Potential Pain, Distress and Treatment of Animal Subjects

All experimental procedures were in compliance with the National Institutes of Health (NIH) Guide for Care and Use of Laboratory Animals and approved by the Institutional Animal Care and Use Committee (IACUC) at the State University of New York at Binghamton. During treatment, behavioral testing, and tissue collection procedures were devised to minimize the potential pain and distress of the animals used in this study. All rats were frequently monitored, at least three times a week, for health status.

**Fig 1:**
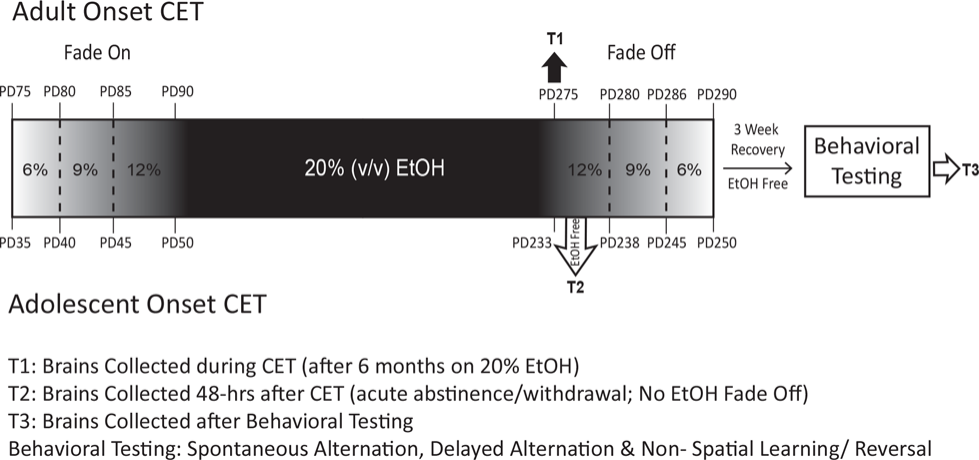
Chronic Ethanol Treatment (CET) Protocol Outline. Schematic illustrating the age range, ethanol (EtOH) exposure procedures, brain collection time points, and behavioral testing paradigms and for both adolescent onset and adult onset CET animals.

Adult (postnatal day [PD] 72–75; n = 40) and adolescent (PD 35; n = 40) male, Sprague-Dawley rats were obtained from litters bred at Binghamton University. Rats were doubly housed in a temperature (20°C) and humidity controlled room under a 12-hour light/dark cycle, 7:00 a.m.-7:00 p.m. No more than one rat from each litter was included in a given treatment condition. Rats were provided with *ad libitum* access to rat chow. Rats were randomly assigned to 1 of 3 tissue collection time-points. At time-point 1 (T1; intoxication), brain tissue was collected during the 28^th^ week of CET while rats were still consuming 20% EtOH. At time-point 2 (T2; acute abstinence or withdrawal), brain tissue was collected 48-hrs after CET ended, in an EtOH-free state. At time-point 3 (T3; protracted abstinence or recovered), brain tissue was collected after behavioral testing, approximately 6–8 weeks following the cessation of CET. Rats were randomly assigned to the following treatment onset conditions: Adolescent control (T1 = 4*, T2 = 4* [*note: T1 and T2 groups were combined into a common control group]; T3 = 8); Adult control (T1 = 4*, T2 = 4*; T3 = 8); Adolescent Onset CET (T1 = 8; T2 = 8; T3 = 8); Adult Onset CET (T1 = 8; T2 = 8; T3 = 8). Fig 1 illustrates the treatment and behavioral timeline for rats undergoing CET and the treatment conditions are defined in detail below.

### Chronic EtOH treatment (CET)

CET began on PD 35 for mid-adolescents and PD 72–75 for adults. Rats exposed to CET were provided with an aqueous solution of EtOH (95% EtOH *v/v*) diluted with tap water to the appropriate *v/v* as the sole source of liquid for the duration of CET. Bottles were weighed and refilled every Monday, Wednesday and Friday. The grams of EtOH per kilogram of body weight was determined by the following formula: (milliliters of EtOH consumed x the percent concentration of EtOH x the density of EtOH/ weight of the animal). Average daily EtOH consumption was determined by dividing the average weekly consumption by seven days across the EtOH exposure phase. CET rats initiated their treatment using a”fading on” procedure [52,57,58]: EtOH exposure started at 6% (*v/v*) for 4 days and incrementally increased by 3% every 5 days until reaching 12% (*v/v*), at which point the concentration was increased to 20% (*v/v)* and maintained for 28 weeks. At the beginning of 20% *(v/v)* exposure, adolescent onset CET rats were PD 50 and adult onset CET rats were approximately PD 90. After 28 weeks, animals in the T3 groups were gradually “faded off” CET in a series of deescalating EtOH concentrations: 12% *(v/v)* for 5 days, 9% *(v/v)* for 5 days, 6% *(v/v)* for 5 days before given *ad libitum* access to water. Control rats were provided with unlimited access to tap water. Water consumption data were collected from age-matched, male, Sprague Dawley rats (S1 Fig).

After 1 month of exposure to 20% EtOH *v/v*, tail blood was collected from all rats at approximately 12:00 AM. This procedure was repeated at months 2, 4, and 6 of CET. Plasma was separated using a centrifuge and blood EtOH concentrations (BEC) were analyzed via Analox (AM1, Analox Instruments, London, United Kingdom). Tail bloods were collected and analyzed from both CET and control groups in order to maintain equal treatment parameters across all animals and in order to validate the BEC assay.

### Brain Collection

Upon rapid decapitation at their given time-points (T1, T2, T3; Fig 1), each rat’s brain was immediately extracted and fresh tissue was extracted from a hemi- dissection. The prefrontal cortex was blocked and collected to include all subregions, and the entire hippocampus was dissected from the hemisphere. Tissue was stored at -80°C to be used for neurotrophin enzyme-linked immunosorbent (ELISA) assays.

### Enzyme-linked immunosorbent assay (ELISA)

The concentration levels of mBDNF (Emax^®^ Immunassay system, Promega, Madison, WI, USA) and β-NGF (Duoset ELISA β-NGF kit, R&D Systems, Minneapolis, MN) were assessed using the respective vendor procedures and protocols. Each region was homogenized, as described by Gearhart and colleagues [59], and returned to the -80°C freezer until ELISA analysis. Total protein concentrations for each region of interest were determined with BCA assay (Pierce ^TM^ BCA Protein Assay Kit, Thermo Scientific, Rockford, IL). The total protein concentration for each neurotrophin and of each sample was calculated using regression analysis based on the standard curve optical densities.

#### Mature BDNF ELISA.

Corning Costar^®^ 96-well flat bottom plates (Corning Life Sciences, Corning, NY) were coated and incubated overnight at 4°C with monoclonal anti-BDNF carbonate buffer (supplied by Promega). Plates were washed in Tris-buffered saline with Tween 20 (TBST) following all incubation periods. The next day, plates were incubated with block and sample buffer for 1-hr at room temperature. Standards (supplied by Promega) were diluted in blocking buffer to create working concentrations of 500, 250, 125, 62.5, 31.5, 15.625, 7.8125, and 0 pg/mL. All samples were diluted at a ratio of 1:4 for the frontal cortex and 1:5 for the hippocampus to allow for an appropriate detection range of the standard curve. Following block, samples were plated and incubated for 2-hrs at room temperature. Samples were then incubated with the supplied BDNF polyclonal antibody for 2-hrs at room temperature. BDNF plates were washed and treated with anti-IgY horseradish peroxidase (HRP) conjugate for 1-hr. After the final wash, a color development reaction was initiated by adding TMB One solution for 10 min at room temperature. The color development reactions were stopped by adding 1 N HCl. Optical densities were measured using a Tecan plate reader (Tecan Infinite M200 Pro, Mannëdorf, Switzerland) and plates were read at a 450 nm wavelength.

#### β-NGF ELISA

Corning Costar^®^ 96-well flat bottom plates were coated and incubated overnight with goat anti-rat β-NGF (R&D systems) at 20°C. Plates were washed with 0.05% Tween-20 in phosphate buffered saline following each incubation. The following day, plates were blocked with BSA reagent diluent for 1-hr at room temperature. Standards (supplied by R&D systems) were diluted in blocking buffer to create working concentrations of: 1000, 500, 250, 125, 62.5, 31.5, 15.625, and 0 pg/mL. Samples were diluted at a ratio of 1:4 for the frontal cortex and 1:5 for the hippocampus to allow for an appropriate detection range of the standard curve. Samples were plated and incubated for 2-hrs at room temperature. Plates were then incubated with a detection antibody for 2-hrs at room temperature, followed by an incubation with streptavidin-HRP for 20 minutes. A color development reaction was initiated by adding vendor supplied H_2_O_2_ and tetramethylbenzidine for 20 minutes at room temperature. The color development reactions were stopped by adding 2 N H_2_SO_4_. Optical densities were measured using a Tecan plate reader at a 450 nm wavelength.

### Behavioral Testing

Following the cessation of CET and the fade-off EtOH procedure, T3 rats began a 3-week EtOH free recovery period. During this time rats were weighed and handled daily. Prior to the start of behavioral testing, rats were food restricted to 90% of their free feed weight over the course of 5 days. Rats were behaviorally tested in the following sequence: spontaneous alternation, delayed alternation, non-spatial discrimination learning, and behavioral flexibility.

#### Spontaneous Alternation

Previous findings indicate that a rat performs optimally on a spontaneous alternation maze task when the animal is slightly food restricted, presumably by increasing their motivation to explore a new environment [60]. Once a rat reached 90% of their free weight, they were tested on a single session of spontaneous alternation. Spontaneous alteration was conducted in a plus maze (105.5 x 14.4 x 15 cm) with clear plastic walls and black painted wooden floors. The testing room was rich in visual cues. To habituate the animal to the testing room, rats were placed in the testing room for 20-min before initiating testing. The rat was then placed into the center of the apparatus, and allowed to explore the maze for 18-min of testing, during which arm entries (all four paws within an arm) were recorded. An alternation was defined as entry into four different arms in overlapping successive sequences of 4 arm entries (e.g., the successive arm entries of A, D, C, B, D, A, C, D, B, D, A, C, D, A, C; the first sequence of ADCB was an alternation, but the following sequence of 4 arm entries DCBD was not). The percent alternation score is equal to the ratio of (actual alternations/possible alternations [trial number-3]) X 100. (For the above data set: 5/(15–3) = .416 X 100 = 41.6%.) This criterion is similar to that used in previous experiments [61–63]. Spontaneous alternation scores were corrected for differences in activity between CET and control groups: Arm entries were only recorded up to the average number of entries made by the adult control group (25 total entries), which had the lowest activity level.

#### Delayed Alternation

After spontaneous alternation testing, rats continued food restriction until they reached 85% of their free feed weight. For the two sessions of delayed alternation testing, a T-maze was used that had clear Plexiglas sidewalls (12 cm high), with two goal arms (55 cm), and a start arm (66 cm). The arms were made of wood, painted black, and the maze was elevated 80 cm from the floor. Transparent Plexiglas guillotine doors separated the start box and the two goal boxes from the choice area. The T-maze was located in the same testing room as the spontaneous alternation maze with the same visual cues. The mazes were placed in the same location so that exposure to spontaneous alternation would also serve as pre-exposure for delayed alternation.

After a 20-min habituation period, the rat was placed in the start box of the maze. On the first trial of each testing day, each rat was allowed a free choice to enter either the left or right arm of the maze, and either response was rewarded with a ½ Frosted Cheerio (General Mills, Minneapolis, MN). After consuming the reward, the rat was manually removed from the goal box and placed back into the start box. There was a 30-sec delay interval between the end of the previous trial and the beginning of the next trial. A correct choice (entering the previously non-visited goal arm) was rewarded with ½ Cheerio. An incorrect response (repeat visit to the previous arm) was not rewarded and the rat was confined to the goal box for a 10-sec time-out period before being returned to the start box. The correct response after an error trial was to alternate to the opposing arm. Percent alternation scores were determined by the number of correct alternations divided by the total number of possible alternations.

#### Non-spatial discrimination learning and Reversal Learning

Following delayed alternation testing, rats began dig training in their home cage for 3 days. Small ceramic bowls (diameter = 9 cm; depth = 4 cm) were filled with wood shavings and baited with ¼ frosted Cheerio. Training began by placing a Cheerio portion on top of the shavings; Cheerios were incrementally placed lower in the shavings until the animal learned to dig the reward out from the bottom of the bowl.

Non-spatial discrimination learning and reversal testing took place in a white opaque plastic box (70.3 x 40 x 36.4 cm) with a black floor. A white, opaque, removable divider sectioned the apparatus into the start box (16.5 x 40) and the testing area (53.8 x 40 cm). Two ceramic bowls, filled with digging substrate termed medium (see Table 1), were located near the back wall separated by another removable divider (19.8 x 25.9 cm). The first phase of training consisted of habituation to the chamber. After 5 min, the divider was lifted allowing the rat to access the baited bowls. When a rat approached the bowls reliably within 30 sec and ate the reward on 6-consecutive trials within 2 min, the rat would advance to discrimination training. This benchmark (a rat approaching and eating from the correct baited bowl on 6 consecutive trials) was used to determine the total number of trials required to reach criterion for the simple, compound and reversal discrimination tasks.

**Table 1:** Non-Spatial Discrimination Reversal Learning Examples

Discrimination Task	Medium-based Cues		Scent-base Cue	
	Bowl 1	Bowl 2	Bowl 1	Bowl 2
Simple	**easter grass**	shredded paper	**clove**/bedding	nutmeg/bedding
Compound 1	thyme/**rocks**	citronella/tubes	piña colada/confetti	**lavender/**colored beads
Reversal 2	thyme/rocks	citronella/**tubes**	**piña colada**/confetti	lavender/colored beads
Compound 2	rosemary/sand	cinnamon/**gravel**	**sweet pea**/sand	vanilla/wood chips
Reversal 2	rosemary/**sand**	cinnamon/gravel	sweet pea/sand	**vanilla**/wood chips

Bolded exemplar indicates the rewarded cue. Half of the animals underwent training with medium-based cues, and the other half underwent training with scent-based cues. The type of exemplar used did not interact with treatment conditions (all *p*’s>0.10).

The second phase of training consisted of each rat learning two simple discriminations: a scent-based and medium-based discrimination. The final phase of testing required each rat to perform a series of five discriminations (see Table 1): (1) Simple discrimination consisted of a single dimension (scent or medium); (2) Compound discrimination included both a unique smell and digging medium that were distinctive from those used in previous discriminations; (3) After reaching criteria on the first compound discrimination, the rule was changed (reversed) such that the previous incorrect combination of stimuli were now correct; (4) A second compound discrimination was trained in which each rat learned a novel complex discrimination with new stimuli (both scent and medium); (5) A final reversal was conducted that switched the rewarded stimuli from the second compound discrimination.

### Experimental Design and Statistics

Analyses were performed in SPSS (IBM Corporation, version 22, Armonk, New York). The neurotrophin data were analyzed using a 2 (Age: adult vs. adolescent) x 2 (Treatment: CET vs. control [water]) x 3 (Time-point of tissue collection: intoxicated [during CET], withdrawal [48-hrs post CET], or protracted recovery [6-8-wks post CET]) factorial analysis of variance. All behavioral data, including both drinking and cognitive assessment, were analyzed with a 2 (Age: adult vs. adolescent) x 2 (Treatment: CET vs. control) factorial analysis of variance. Bivariate correlations were analyzed using Pearson’s correlation coefficient between BECs, neurotrophin level and behavior.

## Results

### Treatment Parameters

#### All rats gained weight across treatment, but CET and Control rats differed at specific time-points

**Fig 2:**
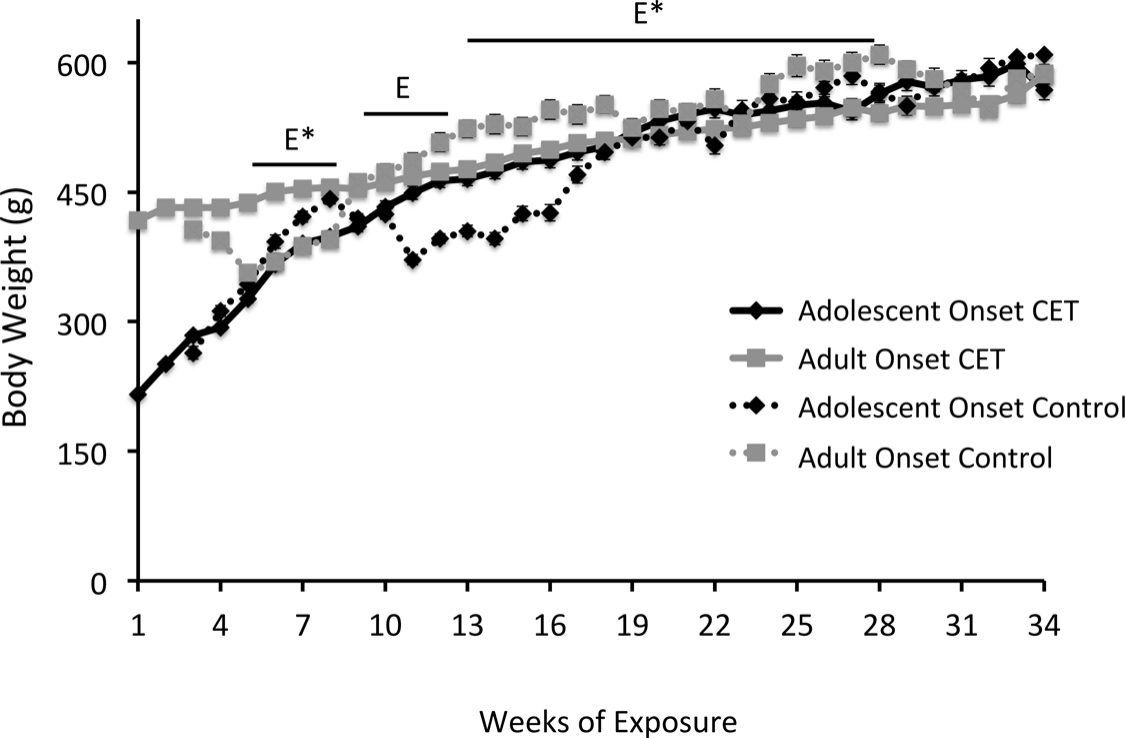
All rats gained weight across treatment, but there were time-points when group weight differences were evident. A significant difference was observed between adolescent onset CET and their age-matched controls during weeks 8–11 [E, *p* <0.05], but not for the remainder of the experiment. During weeks 4–7 and 12–27 [E*, *p* <0.05], a significant difference was also observed between adult onset CET and their age- matched controls.

All rats gained weight (Fig 2) during the course of the experiment (F[27,1431] = 853.44, *p*<0.001). Adolescent onset CET rats weighed significantly more (3.5%) than their age-matched controls (F[1, 38] = 30.63, *p*<0.0001] during weeks 8–11, but no difference was found between these two groups during the remainder of the experiment. Adult onset CET rats weighed significantly more than age-matched controls during weeks 4–7, but adult control rats were significantly heavier (8.4%) than adult onset CET rats from weeks 12–27 (F[1,38] = 6.13, *p*<0.02).

#### Adolescent onset CET rats consumed more EtOH solution

**Fig 3:**
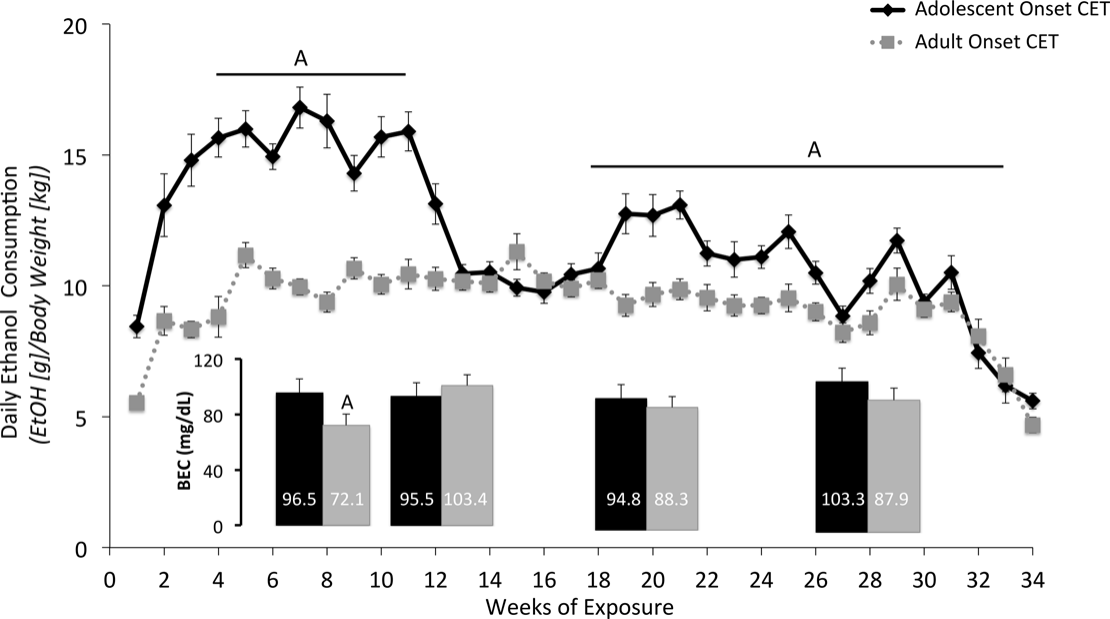
Adolescent onset of drinking, relative to adult onset, leads to higher EtOH consumption levels and initial higher blood ethanol concentrations. A difference in EtOH consumption was observed between the adult and adolescent CET onset rats on weeks 5–11 and 16–31, [A, *p* <0.05], where adolescent onset rats consumed significantly more EtOH solution compared to adult onset rats. Despite significant differences in consumption, a significant difference in BEC was only observed after 1 month of CET [A, *p* <0.05], where adolescent onset rats had significantly higher BEC levels compared to adult onset rats.

As shown in Fig 3, there were age differences in the amount of EtOH consumed during treatment. The average daily EtOH consumption for adult onset rats averaged 9.28 ± 0.61 (SEM) g/kg, whereas adolescent onset rats consumed 11.80 ± 1.11 g/kg. There was a significant interaction between Age and Time (analyzed as weekly intake) on EtOH consumption (F[30,1380] = 11.02, *p*<0.001). Follow-up analyses were conducted by dividing the 34-week treatment phase into 3 (fading-on or fading-off periods) or 4-week bins. Adolescent onset rats drank more than adult onset rats during weeks 4–7 (F[3,138] = 8.83, *p*<0.05), 8-11(F[3,138] = 4.12, *p*<0.05), 16–19 (F[3,138] = 11.63, *p*<0.05), 20–23 (F[3,138] = 6.32, *p*<0.05), 24–27 (F[3,138] = 3.5, *p*<0.05), 28–31 (F[3,138] = 9.62, *p*<0.05). No significant differences were found between weeks 12–15 and 32–34.

As shown in S1 Fig, there was a significant difference in average daily water consumption between adolescent and adult rats during weeks 1–4 (F[3,45] = 3.97, *p*<0.2). Adolescent animals consumed more water compared to adult animals during week 1 (F[1,18] = 87.66, *p<*0.001), week 2 (F[1,15] = 28.80, *p<*0.001), and week 3 (F[1,15] = 12.82, *p<*0.01). After week 4, there was no significant effect of Age on average daily intake (all F’s<2.2, *p’s*>0.05). These results indicate that age-related differences in normal liquid consumption equalize at about P55, the age at which adolescent onset CET animals began exposure to 20% (*v/v*) CET.

#### Rats exposed to chronic EtOH had BECs in the binge range

CET rats had significantly higher BECs than control groups at each analysis time-point (month 2, 3, 4 and 6; F [1,76] = 239.56, *p*<0.001), indicating that control rats were not exposed to EtOH, whereas CET rats were intoxicated. The average BECs across the months were above the binge range of 80 mg/dL (Reilly et al, 2014). A significant Age effect was only found in the month 1 BEC analysis, where adult onset CET rats had lower BECs compared to adolescent onset animals (Fig 3; F[1,47] = 5.13, *p*<0.05).

### Chronic EtOH drinking reduced neurotrophin levels in the prefrontal cortex

#### Brain derived neurotrophin factor

**Fig 4:**
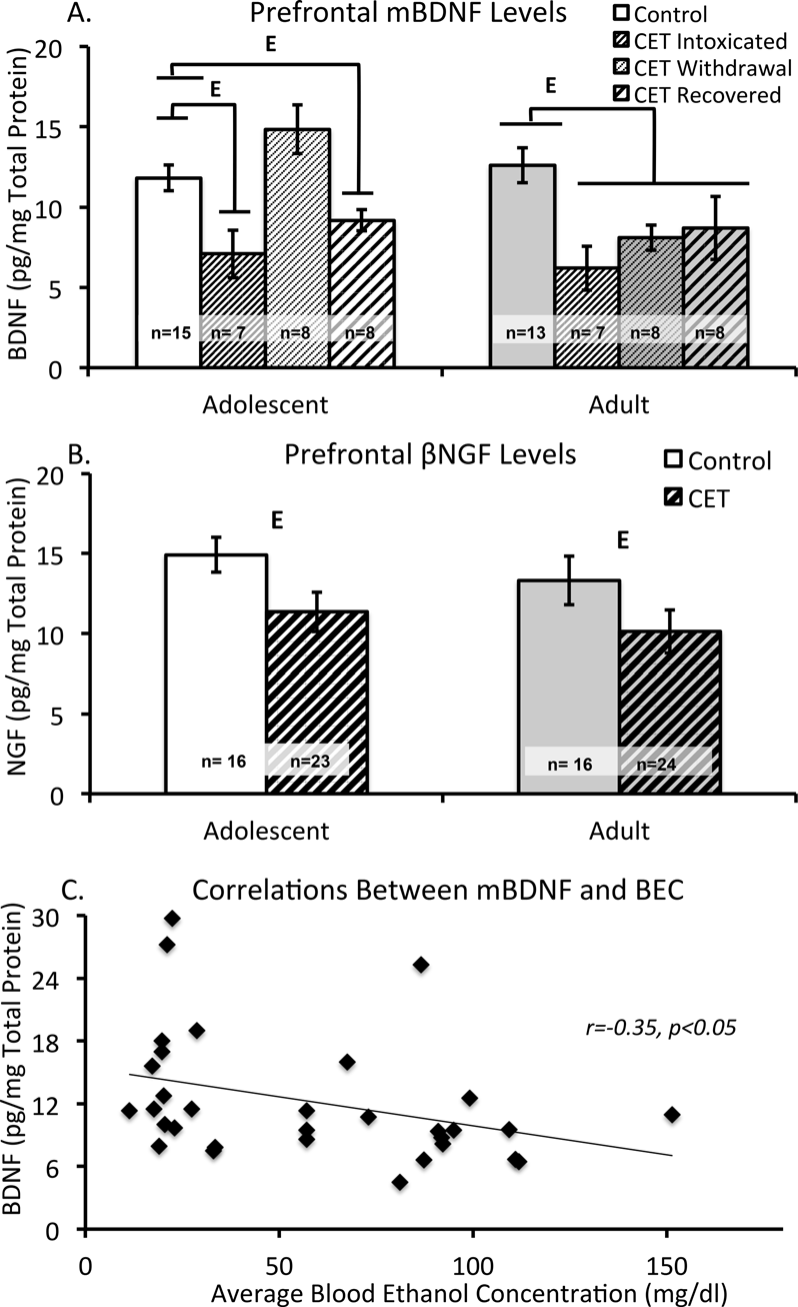
Chronic EtOH exposure alters prefrontal neurotrophin levels. (A) Average concentration of mature BDNF levels (pg/mg) in the prefrontal cortex according to Age, Treatment group and Time-point for tissue collection (control groups were collapsed across time-points). In the adolescent group, time-point 1 [E, p<0.05, intoxicated; thin stripe] and time-point 3 [E, p<0.05, recovered; thick stripe] rats had significantly less BDNF content compared to controls. There was also a significant main effect of Treatment in the adult consumption groups [E, p<0.05], with CET rats having lower BDNF content compared to controls. (B) Mean concentration levels (pg/mg) for β-NGF in the prefrontal cortex by Age and Treatment group, collapsed across time-points. There was a main effect of Treatment [E, p<0.05], with CET rats displaying significantly decreased levels of β-NGF in the prefrontal cortex. (C) Correlation between average BEC levels during CET and BDNF content in the prefrontal cortex. There was a significant negative correlation between average BEC and BDNF content: Rats that had higher average BECs had reduced BDNF levels within the prefrontal cortex.

The cortices of 5 rats (1- Adolescent CET/T1; 1- Adolescent control/T3; 1-Adult CET/T1; 2-Adult Control T1) were not processed due to limited tissue. We hypothesized that there would be no differences across time-points for neurotrophin content in the control groups. This hypothesis was supported by the ANOVA (both F’s <1.21, *p*>0.30), allowing us to collapse our data across time-points and use a single control group at each age. There was a main effect of Treatment (CET vs. Control) on prefrontal cortex BDNF levels (F[1,66] = 21.29, *p*<0.0001), where CET rats had lower BDNF levels than controls rats. In the CET groups, there was significant Age x Time interaction (F[2,66] = 3.55, *p*<0.05). As shown in Fig 4A, adolescent onset CET rats at T1 (intoxicated) and T3 (protracted abstinence/recovered) had significantly decreased BDNF levels relative to control rats (T1: F[1,21] = 9.27, *p*<0.05; T3: F[1,22] = 4.76, *p*<0.05). At T2 (acute abstinence/withdrawal) there were was a trend (F[1,21] = 3.75, *p* = 0.07) for adolescent onset CET rats to have higher BDNF levels than control rats, but this difference failed to reach significance. In contrast, the adult onset CET rats had suppressed BDNF levels, relative to their control group, at all time points (F[1,32] = 11.66, *p*<0.01). Unlike the adolescent CET rats, adult CET rats did not differ across the three time points (F<1).

#### β-Nerve growth factor

The cortex of 1 rat (1-Adolescent CET/T2) was not processed due to limited tissue availability. There were no age related differences in prefrontal cortex β-NGF levels in control (both F’s<1.76, *p’s*>0.20) or CET groups (both F’s<1.13, *p’s*>0.30) across the 3 time-points. Therefore, the data were collapsed across time points for both treatment groups. A main effect of Treatment was found (F[1,71] = 6.01, *p*<0.05), with CET rats having lower levels of β-NGF (Fig 4B). However, there was no main effect of Age (F’s< 0.58, *p’s* >0.451, or Time point (F’s< 1.31, *p’s* >0.28) and the interaction between those variables was non-significant (F’s< 1.3, *p*’s> 0.28).

#### Higher BECs correlated with lower prefrontal cortical BDNF levels

Regardless of the age at which EtOH exposure initiated, rats with higher BECs had decreased BDNF content within the prefrontal cortex (Fig 4C). Average BEC levels had a significant, negative correlation with prefrontal BDNF content, r = -0.35, *p*<0.05. This was the only regional neurotrophin level that correlated with BEC.

### Chronic EtOH drinking reduces hippocampal BDNF levels in adult onset rats

#### Brain derived neurotrophin factor

**Fig 5:**
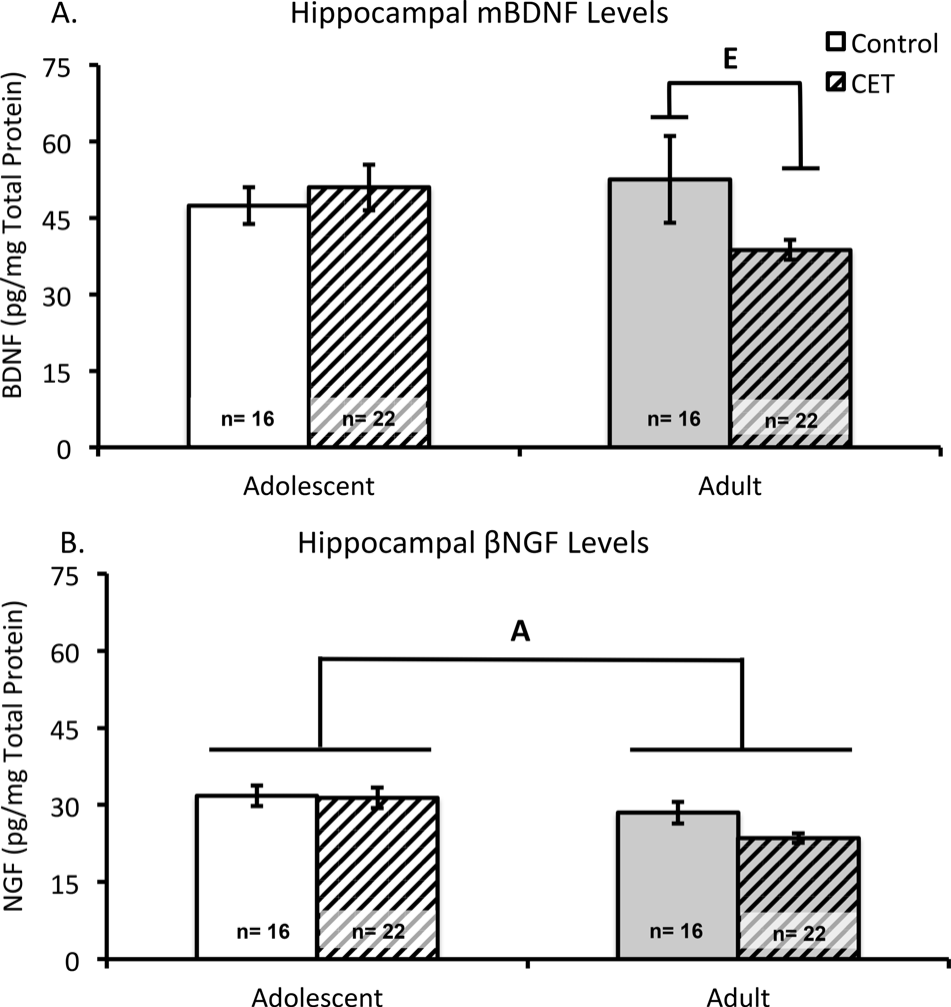
Chronic EtOH drinking decreases hippocampal neurotrophin levels only in adult onset EtOH drinking rats. (A) Mean concentration of mature BDNF levels (pg/mg) in the hippocampus according to Age and Treatment conditions, collapsed across time-points for tissue collection. Adult CET rats have less BDNF neurotrophin content compared to adult controls [E, *p* <0.05]. (B) Mean concentration levels of β-NGF (pg/mg) in the hippocampus according to Age and Treatment groups, collapsed across time-points for tissue collection. There was a main effect of Age [A, p<0.05] on β-NGF levels, where adult rats have significantly less β-NGF detected in the hippocampus compared to adolescent rats.

The hippocampi of 4 subjects (1-Adolescent CET/T1; 1-Adolescent CET/T2; 2 Adult CET/T2) were not processed due to dissection issues or tissue availability. Neither adolescent nor adult rats, CET nor control, significantly differed in hippocampal BDNF levels as a function of Time point (F’s<1, p’s> 0.60); thus, data were collapsed across time-points (Fig 5A). Analysis of variance revealed a trend towards significance in the interaction between Age and Treatment (F(1,68) = 3.79, *p* = 0.056). Post-hoc analyses revealed that adult onset CET rats had significantly lower hippocampal BDNF levels than adult control rats (F[1,36] = 4.16, *p*<0.05). This treatment effect was not observed between the adolescent onset rats (F<1).

#### β-Nerve Growth Factor

Both control and CET groups were collapsed across time-points as levels of β-NGF in the hippocampus did not differ as a function of time-points (F’s<1, p’s>0.90). Regardless of treatment condition, animals that began the exposure protocol as adolescents had significantly higher concentrations of β-NGF in the hippocampus than their adult counterparts (F[1,68] = 9.48, *p*<0.01; Fig 5B). There was no significant interaction between Age and Treatment (F[1,72] = 1.65, p>0.20).

### Behavioral Testing

All T-3 groups (Adolescent onset CET, Adolescent water control, Adult onset CET, Adult water control) had 8 subjects that completed all phases of behavioral testing.

#### Higher BECs correlated with lower spontaneous alternation scores

**Fig 6:**
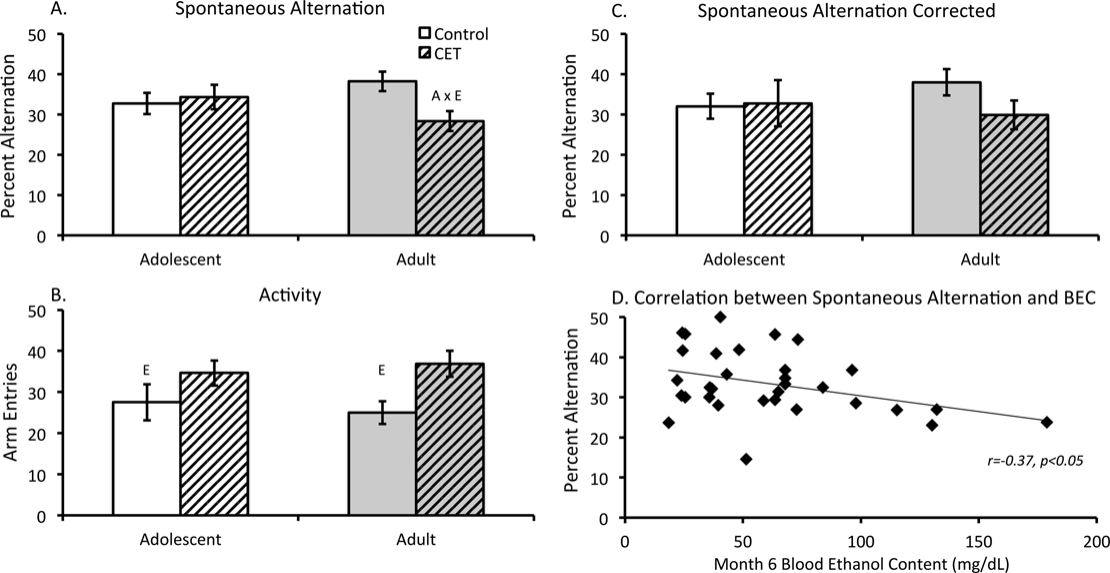
Lower spontaneous alternation behavior correlated with higher blood EtOH concentrations. (A) Percent alternation analysis reveals an Age by Treatment interaction [A x E, p<0.05], with adult onset CET rats alternating significantly less than age-matched controls. (B) Mean number of arm entries according to Age and Treatment groups. Regardless of age, CET rats made significantly more arm entries compared to controls. (C) Analysis of percent alteration scores corrected for a maximum of 25 arm entries revealed no difference between adult control and CET rats. (D) Final (month 6) BECs were significantly negatively correlated with spontaneous alternation behavior: As BECs increased, spontaneous alternation performance decreased.

Analysis of the percent alternation scores revealed a significant Age x Treatment interaction (F [1,28] = 4.66, *p*<0.05; Fig 6A). Adult onset CET rats displayed lower alternation scores compared to their age-matched controls. In contrast, adolescent onset CET rats did not display significantly different alternation scores relative to age-matched controls. However, CET rats, regardless of age, made significantly more arm entries compared to control rats (F [1, 28] = 7.77, *p*<0.01; Fig 6B). Thus, alternation scores were corrected for overall activity, with percent alternation scores only including a maximum of 25 arm entries (the lowest group activity level). Subsequent analysis failed to yield a significant difference between adult onset CET and the adult control group (F[1,14) = 2.95, *p* = 0.11; (Fig 6C).

However, the correlation analysis revealed a negative relation (r = -0.37, *p*<0.05) between high BECs and low spontaneous alternation scores (Fig 6D). Regardless of the age at which EtOH exposure initiated, rats with higher BECs at the end of the CET paradigm had significantly fewer spontaneous alternations.

#### Delayed Alternation is not affected by chronic EtOH drinking

**Fig 7:**
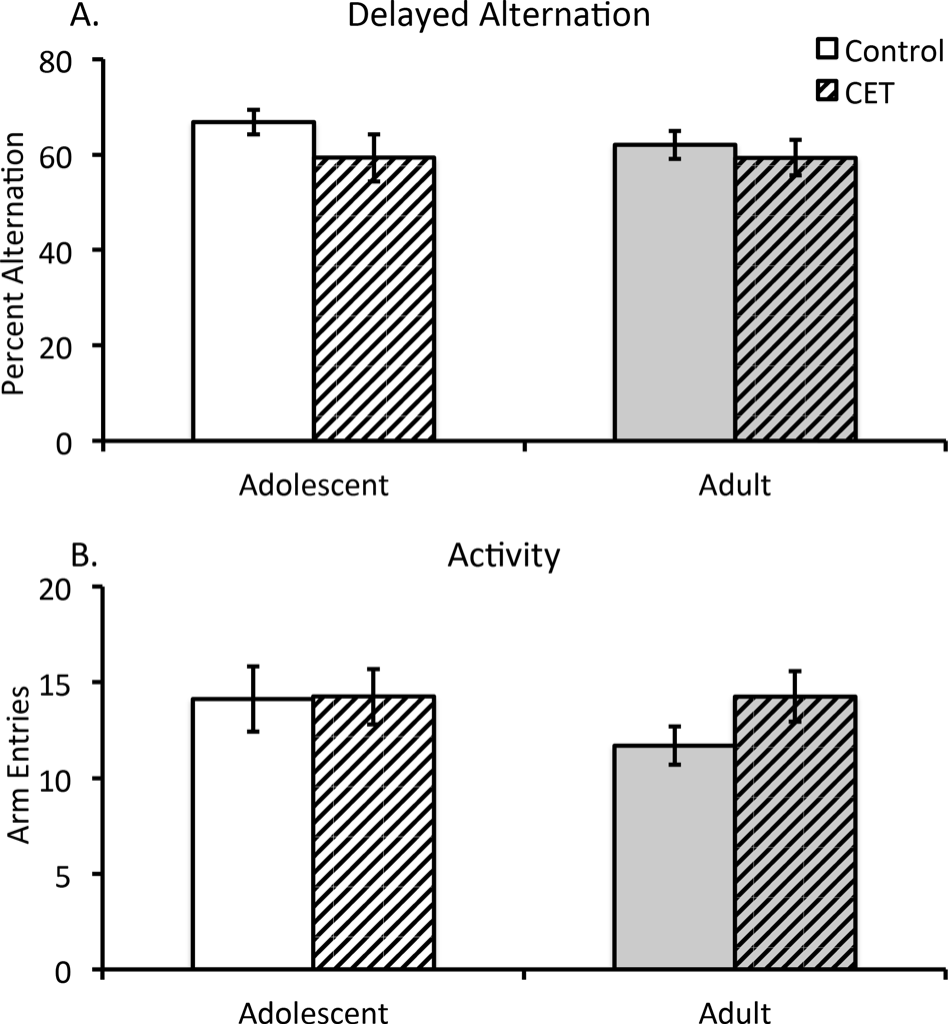
Chronic EtOH drinking did not impair delayed alternation behavior. (A) Average percent delayed alternation according to Age and Treatment conditions. There was no effect of Age or CET on delayed alternation performance. (B) Average number of arm entries according to Age and Treatment groups. No group differences were found.

There were no significant differences in delayed alternation scores or arm entries as a function of Age or Treatment (F’s[1,28) <1.73; *p*>0.10). Furthermore, there was no interaction between Age and Treatment on delayed alternation scores (All F’s <0.25, *p’s*>0.05; Fig 7).

#### Chronic EtOH drinking impairs non-spatial discrimination learning and behavioral flexibility

**Fig 8:**
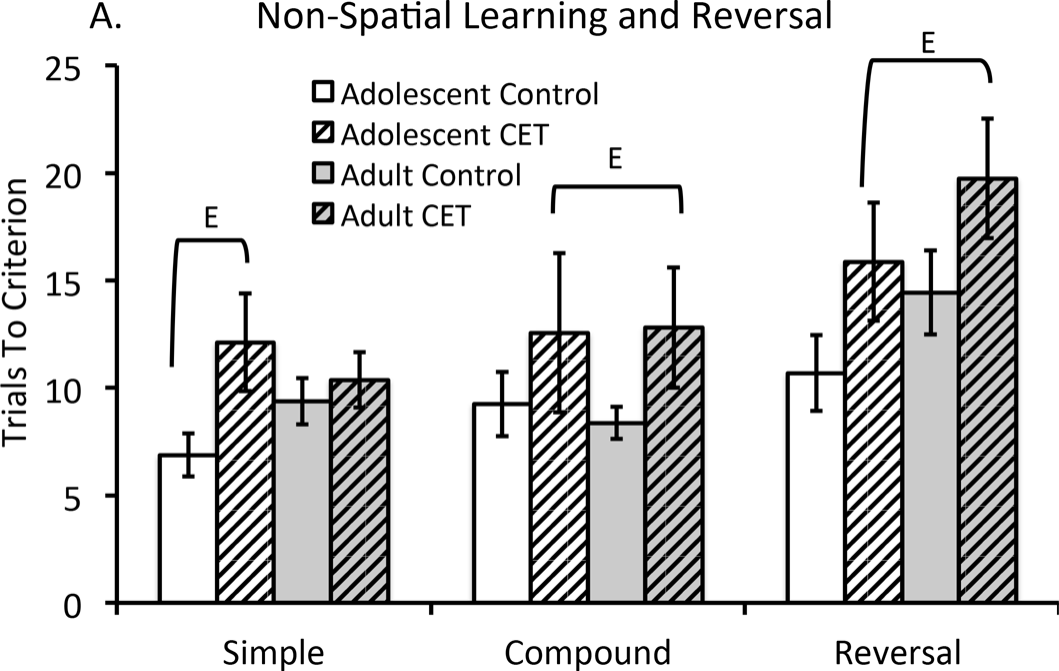
Chronic EtOH drinking, regardless of age of drinking onset, impaired complex non-spatial discrimination learning and reduced behavioral flexibility. (A) The average number of trials required to reach criterion during non-spatial discrimination learning and reversal tasks. Criterion was defined as making a correct discrimination on 6 consecutive trials. Significant CET effects [E] were found on the simple discrimination task, compound discrimination and reversal phases. In these three tasks, CET rats all required more trials to reach criterion compared to controls (E, p’s<0.05).

Fig 8A demonstrates the average number of trials required to reach criterion as a function of the type of non-spatial discrimination task for each group. The number of trials to criterion varied as a function of test (F[4,112] = 12.96, *p*<0.001). There was a main effect of CET that demonstrated impaired non-spatial discrimination learning, (F[1,28] = 8.82, *p*<0.01), but EtOH exposure interacted with the type of task (F[4,112] = 2.99, *p*<0.025). We therefore analyzed separate ANOVAs for each type of task. This analysis revealed that CET rats required more trials to learn the simple discrimination (F [1, 28] = 4.33, *p*<0.05). However, the effect was driven by the CET adolescent group requiring more trials to master the simple discrimination rule (F[1,28] = 4.65, *p*<0.05), whereas adult CET rats did not differ from adult controls (F<1). Since there was no significant difference across both compound (F[1,28] = 3.36, *p*>0.05) and reversal tasks (F[1,28] = 1.55, *p*>0.05), each test was collapsed. Both CET age groups required more trials to learn the complex discriminations (F[1, 28] = 4.93, *p*<0.05). Furthermore, the greatest deficit (Cohen’s *d* = 0.79) was seen in reversal learning, where CET rats were impaired on reversal learning regardless of the age of onset (F[1, 28] = 7.27, *p*<0.025).

We did not find a significant correlation between BEC levels at the end of CET and the number of trials to reach criterion for non-spatial discrimination learning or reversal learning (all *p*’s>0.05).

## Discussion

Four main findings emerged from the present study: first, adolescent onset of chronic EtOH drinking led to increased EtOH consumption that persisted beyond the adolescent period into adulthood. Second, chronic EtOH drinking altered both brain BDNF and NGF levels during intoxication, withdrawal and protracted abstinence. In the prefrontal cortex, CET produced long-term decreases in both BDNF and NGF, regardless of the age at which EtOH consumption was initiated. However, adolescent onset CET rats displayed a unique increase in BDNF levels during the acute abstinence (withdrawal) phase. In the hippocampus, only rats that started consuming EtOH as adults displayed reductions in BDNF. Third, higher BECs were correlated with lower hippocampal-dependent spontaneous alternation behavior. Fourth, regardless of BECs or age of drinking onset, non-spatial discrimination learning and behavioral flexibility, which is dependent on the frontal cortex, was impaired by chronic EtOH drinking.

Epidemiological work has consistently demonstrated that adolescent alcohol abuse increases the lifetime risk for developing a later AUD [9,64,65]. This has been mirrored in animal models demonstrating that adolescent EtOH exposure predisposes rats to consume elevated levels of EtOH in adulthood, compared to an EtOH naïve rat [66–71]. Rats with an adolescent onset of CET had comparable BEC levels to adult onset CET rats after month 1, despite the fact that adult onset CET rats consumed, on average, 10% less EtOH. The data suggest a greater metabolic tolerance in the adolescent onset CET group relative to the adult onset ETOH group. This finding complements data showing that adolescent rats exposed to a chronic, intermittent binge EtOH treatment display an increased metabolic rate, relative to adult rats [72]. The comparable BEC levels across the adolescent and adult onset CET groups likely contributed to the similar profile of behavioral impairment seen in both exposure groups.

Our CET animals exhibited impairments in non- spatial discrimination learning, as well as overall reductions in neurotrophin levels. It is hypothesized that alcohol-related brain damage, leading to behavioral dysfunction, is a result of alterations in the balance between neurotrophins, which are reduced, and neuroimmune signaling, which is typically increased, after chronic EtOH exposure [48,73]. At high doses, EtOH decreases cyclic AMP-responsive element binding protein (CREB)-DNA binding while increasing nuclear factor kappa-light-chain enhancer of activated B cells (NF-kB; [74]). EtOH-induced disruptions of CREB and NF-kB levels may result in decreased neurotrophin levels and increased chemokine and cytokine activation, which exacerbate EtOH-induced glutamate excitoxicity, which could manifest in behavioral deficits [48].

Neuroimaging studies of the alcoholic brain have demonstrated that the frontal lobes, relative to other brain regions, have the most pronounced abnormalities [75,76]. These findings are paralleled by an increase in markers of cell death and neural neuroinflammation within the frontal cortex in both rodent models of adolescent binge EtOH exposure and in post mortem brain tissue of alcoholics with an early age of drinking onset [26]. Abstinent alcoholics display decreased gray matter volume in the dorsolateral, dorsomedial and ventromedial prefrontal cortex that are correlated with increased impulsivity [77]. Orbital frontal cortical shrinkage is also observed in abstinent alcoholics [78] and has been associated with impaired behavioral flexibility [37], including reversal learning [79]. Similarly, rodents exposed to chronic intermittent EtOH exposure as adults [80] or adolescents [26] display an initial impairment in reversal learning, but in adult mice the reversal impairment was no longer evident with an extended recovery period (10-days post EtOH). Our data demonstrate that long-term, continuous EtOH exposure leads to a persistent and long-lasting impairment in reversal learning, regardless of age of drinking onset or BEC.

However, brain and behavioral recovery following abstinence from alcohol consumption has been observed in humans, but there are considerable individual differences [81]. Following excessive binge EtOH exposure in rodents, in the acute abstinence phase (48-hrs post EtOH), there is a burst of cell genesis and an increase in phosphorylated CREB in multiple brain regions, including the cortex and hippocampus [82]. However, the increase in these markers of plasticity eventually subsides. Alcoholic patients going through withdrawal show an acute transient increase in plasma concentrations of BDNF levels, but not NGF levels [83,84]. Furthermore, the intensity of acute alcohol withdrawal symptoms have been correlated with an increase in BDNF serum levels [46], which was taken as evidence that BDNF may be involved in neuroadaptation during the early alcohol withdrawal period. The transient increase in frontal cortical mBDNF observed in the current study (during the acute abstinence phase in adolescent onset CET rats) may represent an attempt to compensate for neurodegeneration. This effect could be indicative of an intrinsic mechanism for the repair of alcohol-induced structural damage, similar to what has been observed after adolescent binge EtOH exposure [20]. Age at the time of EtOH exposure maybe a key factor in both the expression and extent of the recovery of function observed in abstinent alcoholics. The manner in which age and alcohol interact to modulate both neural adaption and plasticity markers needs further examination.

In the adult nervous system, the mature forms of BDNF and NGF support neuronal survival and are critical for synaptic plasticity that is altered by addiction [85]. However, both pro-NGF and pro-BDNF activate the p75 receptor that leads to cell death [86,87]. Chronic, continuous EtOH exposure decreases the mRNA for BDNF within the adult cortex during CET [88] and within the hippocampus 48-hrs after EtOH cessation [89]. The down-regulation of BDNF transcription, induced by CET, could lead to the reduction of BDNF synthesis, thereby resulting in decreased detection of mBDNF levels after prolonged EtOH consumption. Chronic EtOH intake in rats also decreases the immunoreactivity of NGF and choline acetyltransferase, an enzyme regulated by NGF, within the hippocampus and medial septum [51]. This suggests that NGF synthesis and/or biological activity is also affected by chronic EtOH drinking. This is supported by studies demonstrating that exogenous NGF delivery recovers both behavioral impairment and the loss of forebrain cholinergic neurons that project to the hippocampus and cortex in the CET model [53,54].

We observed a significant loss of NGF in the frontal cortex, regardless of age of drinking onset, an effect not observed in the hippocampus. This regional sensitivity may be attributed to the fact that most ELISA kits measure both the pro- and mature forms of NGF [90]. Considering the opposing effects of pro and mature forms of NGF and BDNF on cell survival, it is critical in future studies to determine the ratio between pro and mature neurotrophins. The altered balance across pro- and mature neurotrophin levels could be a biomarker of neurodegeneration or repair in several diseases, including chronic alcoholism.

Behavioral deficits on tasks that rely on the hippocampus and/or frontal cortex are seen in abstinent alcoholics [91,92] and rodent models of chronic continuous or intermittent EtOH exposure [93]. Factors that contribute to the extent of both hippocampal and cortical impairment are drinking history duration and degree of intoxication, which can be reflected in BECs. It has been stated that a high volume of alcohol drinking (35 drinks per week for men; 28 drinks per week for women) for an extended period of time (more than 5 years) are key risk factors in the development of alcohol-related brain damage [94]. The forced EtOH consumption models (EtOH in the drinking fluid or liquid diet) are analogs of the later stages of sustained alcohol addiction [50]. Such forced EtOH consumption models are commonly used to achieve high EtOH intake for extended periods of time [49], which appear to modulate alcohol-induced behavioral dysfunction.

Spatial memory impairments have been observed after adult onset CET with a long duration (12 months) of EtOH exposure [95] or with shorter durations (6–8 months) when BEC are over 100 mg/dl [96]. Sex also appears to modulate the behavioral effects of chronic EtOH: we found that adult female rats exposed to CET were impaired on delayed matching to position, whereas adult male CET rats only displayed impairment when the rule was reversed to non-matching-to-position [57]. In the current study, we found that higher BECs at the end of treatment were correlated with lower spontaneous alternation performance, a hippocampal dependent task [97]. However, our data reveal that the same correlation between BEC and reversal learning, an orbital frontal cortical dependent task [98], was not significant. Regardless, there was a main effect of CET on reversal learning, irrespective of age of drinking onset. This suggests that EtOH-induced abnormalities in frontocortical-dependent behaviors can be found across a range of metabolic EtOH concentrations. Thus, frontocortical behaviors, like neural plasticity measures, appear to be more sensitive to EtOH, compared to hippocampal-dependent behaviors.

## Conclusions

Recent data establishes adolescence as a vulnerable time period for EtOH related toxicity that leads to neuropathological, behavioral and motivational changes [99]. Initiating alcohol drinking at a young age leads to a greater propensity for the development of AUDs. Individuals who begin drinking by the age of 13 are over 3 times more likely to engage binge drinking (more than 5 drinks per episode) and extreme drinking (greater than 10 drinks per occasion; [100]. Exposure to EtOH during adolescence in rodents can produce greater EtOH intake during adulthood [101,102] and we replicated those finding in a continuous access model.

However, adolescence is a protracted period of developmental neural adaption that appears to have epochs of vulnerability to alcohol exposure, which influences certain long-term behavioral outcomes, including abuse propensity [103]. In rodents, researchers have identified three adolescent epochs: early- (PD 21–34), mid- (PD 34–46), and late- (PD 46–59) adolescence [103,104]. In a summary of recent data, Spear [103] suggests that early adolescent EtOH exposure has been associated with affective and hippocampal abnormalities, whereas late adolescent EtOH exposure disrupts behaviors dependent on the developing pre-frontal cortical systems.

The lack of persistent hippocampal dysfunction after mid-adolescent onset CET may be related to the unique, time- dependent, developmental windows that occur during adolescence. One caveat of our model is that the EtOH “fade on” period takes the adolescent rats through “emerging adulthood” [99]. Thus, the impact of the moderate to high EtOH doses did not occur until emerging adulthood. Exposure to binge EtOH levels during early adolescence have been linked to deficits in spatial memory acquisition and reversal learning [105,106]. These EtOH related deficits are either not elicited or less pronounced when exposure occurs during mid to late adolescence [103]. Therefore, we hypothesize that the lack of age- dependent, EtOH related behavioral deficits was possibly due to the fact that we missed a critical window of EtOH exposure. If EtOH exposure was initiated at an earlier age, it is possible that we would have detected pronounced behavioral and neural deficits compared to those seen with adult onset exposure.

However, even moderate drinking during mid-adolescence contributed to heavy EtOH consumption in adulthood. It has been shown, and we replicated, that by PD 55, water consumption stabilizes across adulthood [107]. Thus, differences in EtOH consumption from day 55 onward are not due to adolescent-typical hyperdipsia.

The current data extend our understanding of the different patterns of neuroadaptation, specifically of BDNF and NGF, within two key brain regions (hippocampus and frontal cortex) in an animal model of late-stage sustained alcohol addiction. By collecting brain samples at different phases of the addiction cycle (intoxication, acute and protracted abstinence), we revealed that chronic EtOH exposure beginning in mid-adolescence or early adulthood leads to reductions in frontocortical plasticity during intoxication and following protracted abstinence. The neurotrophin reductions that occur during active drinking and protracted abstinence may modulate several disease process associated with alcoholism, including both functional (decision making) and structural (neuropathological) changes that likely contribute to the cycle of addictive behaviors [108].

## Supporting Information

S1 Data Behavioral and neurotrophin data. Data is presented for each Treatment condition as a function of age (Age of Onset) at which drinking started (postnatal day 35 = Adolescent; postnatal day 73–75 = Adult), and time (Timepoint) when brains were collected (1 = during ethanol consumption; 2 = 48-hrs after ethanol removal; 3 = 6–8 weeks after ethanol fading-off and behavioral testing). Data is present for each behavioral task (DA = Delayed Alternation [Day 1; Day 2]; SA = Spontaneous Alternation; % = Percent alternation; # = Number of arm entries). Discrimination data is summarized as the number of trials to reach criterion. There is also neurotrophin data (Brain-Derived Neurotropic Factor [BDNF] and Nerve Growth Factor [NGF], both presented as pg/mg of protein, as a function of brain region (PFC = prefrontal cortex; HPC = hippocampus).
(PDF)

S2 Data Estimated daily ethanol consumption. Estimated daily EtOH consumption (g/kg) as a function of ethanol concentration (6%, 9%, 12%, 20%) for 28 weeks for rats that started drinking at either postnatal day 35 (see Age at Onset; Adolescent) or postnatal days 73–75 (see Age at Onset; Adult). Brains were collected at 3 time points (Timepoint): 1 = During ethanol consumption; 2 = 48-hours after ethanol removal; 3 = 6 to 8 weeks post ethanol exposure and after behavioral testing.
(PDF)

S1 Fig Adolescents initially have higher water consumption compared to adults, but this difference disappears by emerging adulthood. A difference in water consumption was observed between adult and adolescent age- matched animals on weeks 1–4 [A, *p* <0.05], where adolescent animals consumed significantly more liquid compared to adult age- matched rats. No further age dependent differences were found on consumption levels after week 4.
(TIF)
