# Supplementary material for: Chronic Drinking During Adolescence Predisposes the Adult Rat for Continued Heavy Drinking: Neurotrophin and Behavioral Adaptation after Long-Term, Continuous Ethanol Exposure
Source: PLoS One. 2016 Mar 1;11(3):e0149987. doi: 10.1371/journal.pone.0149987 (PMC4773001; doi:10.1371/journal.pone.0149987)
Supplement: S1 Data — Data is presented for each Treatment condition as a function of age (Age of Onset) at which drinking started (postnatal day 35 = Adolescent; postnatal day 73–75 = Adult), and time (Timepoint) when brains were collected (1 = during ethanol consumption; 2 = 48-hrs after ethanol removal; 3 = 6–8 weeks after ethanol fading-off and behavioral testing). Data is present for each behavioral task (DA = Delayed Alternation [Day 1; Day 2]; SA = Spontaneous Alternation; % = Percent alternation; # = Number of arm entries). Discrimination data is summarized as the number of trials to reach criterion. There is also neurotrophin data (Brain-Derived Neurotropic Factor [BDNF] and Nerve Growth Factor [NGF], both presented as pg/mg of protein, as a function of brain region (PFC = prefrontal cortex; HPC = hippocampus). (PDF) [file pone.0149987.s001.pdf]

Supplementary Data set 1.

Rat ID	Treatment	Age of Onset	Timepoint	DA1%	DA2%	DA1#	DA2#	SA%	SA#	R-SA	Simple	Compound 1	Reversal 1	Compound 2	Reversal 2	PFC BDNF	PFC NGF	HPC BDNF	HPC-NGF
11	Chronic EtOH Treated	Adolescent	1													2.95	21.45		
17	Chronic EtOH Treated	Adolescent	1													12.24	6.77	35.79	31.53
25	Chronic EtOH Treated	Adolescent	1													4.56	13.24	35.25	30.9
31	Chronic EtOH Treated	Adolescent	1													11.31	10.47	61.05	32.91
39	Chronic EtOH Treated	Adolescent	1													5.66	6.99	81.68	39.67
45	Chronic EtOH Treated	Adolescent	1													3.02	10.21	39.25	25.51
52	Chronic EtOH Treated	Adolescent	1														21.23	58.36	31.57
91	Chronic EtOH Treated	Adolescent	1													9.83	8.68	51.7	32.77
14	Water Treatment Control	Adolescent	1													17.22	11.43	27.73	25.62
16	Water Treatment Control	Adolescent	1													14.94	15.35	59.08	31.33
20	Water Treatment Control	Adolescent	1													13.72	7.55	59.87	38.19
22	Water Treatment Control	Adolescent	1													12.48	16.53	33.74	34.28
26	Water Treatment Control	Adolescent	2													10.71	23.63	62.61	31.04
28	Water Treatment Control	Adolescent	2													9.31	21.16	80.46	32.33
32	Water Treatment Control	Adolescent	2													9.44	11.44	52.72	55.59
34	Water Treatment Control	Adolescent	2													15.98	20.16	50.03	34.54
4	Chronic EtOH Treated	Adult	1													6.77	2.22	60.67	21.18
6	Chronic EtOH Treated	Adult	1													0.76	15.31	51.64	19.2
9	Chronic EtOH Treated	Adult	1													4.57	1.9	25.5	23.88
59	Chronic EtOH Treated	Adult	1													3.97	7.78	30.94	30.08
65	Chronic EtOH Treated	Adult	1													5.93	4.8	43.68	26.49
71	Chronic EtOH Treated	Adult	1													11.03	2.44	42.77	22.12
83	Chronic EtOH Treated	Adult	1													10.31	3.9	28.11	20.32
58	Water Treatment Control	Adult	1													10.98	8.79	39.39	25.3
64	Water Treatment Control	Adult	1													8.47	10.12	38.13	27.35
70	Water Treatment Control	Adult	1													9.93	11.03	142.39	39.97
72	Water Treatment Control	Adult	1													13.85	4.32	24.9	30
78	Water Treatment Control	Adult	1													16.6	14.24	41.68	22.17
82	Water Treatment Control	Adult	2													12.75	10.97	77.09	39.99
84	Water Treatment Control	Adult	2														21.02	33.63	17.19
85	Water Treatment Control	Adult	2													9.68	24.63	50.79	42.09
86	Water Treatment Control	Adult	2													7.51	17.6	109.25	40.94
13	Chronic EtOH Treated	Adolescent	2													15.42	4.67	106.05	30.59
19	Chronic EtOH Treated	Adolescent	2													14.38	12.4		
21	Chronic EtOH Treated	Adolescent	2													11.02	11.65	27.93	23.58
27	Chronic EtOH Treated	Adolescent	2													7.01	5.69	27.07	22.27
33	Chronic EtOH Treated	Adolescent	2													17.43	9.67	85.87	49.89
41	Chronic EtOH Treated	Adolescent	2													14.24	11.32	35.2	24.74
46	Chronic EtOH Treated	Adolescent	2													20.13		46.61	22.39
48	Chronic EtOH Treated	Adolescent	2													19.12	22.72	49.79	40.93
1	Chronic EtOH Treated	Adult	2													10.45	9.94	33.27	23.27
5	Chronic EtOH Treated	Adult	2													5.19	5.88	27.64	20.19
69	Chronic EtOH Treated	Adult	2													11.01	5.98	38.38	23.51
73	Chronic EtOH Treated	Adult	2													10.11	14.92	31.78	25.95
77	Chronic EtOH Treated	Adult	2													7.77	5.67	50.42	25.5
79	Chronic EtOH Treated	Adult	2													7.95	3.47	31.05	24.17
87	Chronic EtOH Treated	Adult	2													5.37	20.78	57.13	
90	Chronic EtOH Treated	Adult	2													6.94	13.25		
15	Chronic EtOH Treated	Adolescent	3	18.75	22.22	17	19	45.71	38	54.5	9	9	18	11	21	10.7	4.92		53.83
23	Chronic EtOH Treated	Adolescent	3	61.54	73.33	14	16	33.33	21	33	11	11	8	6	7	9.33	5.78	65.33	27.43
29	Chronic EtOH Treated	Adolescent	3	72.73	50	12	23	41.86	46	40.9	26	31	34	18	32	12.54	23.86	53.32	33.81
35	Chronic EtOH Treated	Adolescent	3	46.15	83.33	14	7	26.92	29	18.18	6	7	15	6	6	8.77	9.54	40.66	40.09
37	Chronic EtOH Treated	Adolescent	3	71.43	55.55	8	10	44.44	30	50	11	6	21	6	10	6.44	15.62	57.13	10.72
43	Chronic EtOH Treated	Adolescent	3	87.5	50	9	9	31.43	38	31.8	6	11	13	15	8	7.97	19.82	29.13	27.36
44	Chronic EtOH Treated	Adolescent	3	83.33	59.09	7	23	23.81	45	9.09	15	8	18	6	10	8.17	17	45.43	28.01
50	Chronic EtOH Treated	Adolescent	3	53.33	60.8	16	24	26.92	30	22.7	13	32	13	18	20	9.48	2.31	37.87	29.92
18	Water Treatment Control	Adolescent	3	66.66	62.5	10	9	46.15	16	46.15	6	11	11	6	8	11.31	11.02	42.2	24.56
24	Water Treatment Control	Adolescent	3	80	53.85	16	14	28	45	31.8	6	13	9	8	6	7.96	16.05	36.66	31.12
30	Water Treatment Control	Adolescent	3	61.11	58.82	19	18	30	13	30	13	6	21	6	6	8.07	12.19	44.74	28.17

49	Water Treatment Control	Adolescent	3	69.23	87.5	14	9	29.16	27	31.8	6	7	17	7	11	7.82	11.01	34.84	22.03
51	Water Treatment Control	Adolescent	3	62.5	62.5	9	9	23.68	38	18.18	6	19	19	9	11	15.62	15.11	48.14	29.78
88	Water Treatment Control	Adolescent	3	77.78	81.81	10	12	30.43	26	31.8	6	6	7	6	10	9.99	11.31	30.55	26.69
92	Water Treatment Control	Adolescent	3	50	60	9	11	41.66	15	41.6	6	8	7	6	6	12.76	17.96	59.17	24.66
95	Water Treatment Control	Adolescent	3	68.18	66.66	23	34	32.43	40	27.3	6	18	16	12	6		16.84	36.11	37.83
3	Chronic EtOH Treated	Adult	3	56.25	72.22	17	18	23.08	42	27.3	6	10	11	6	10	19.3	13.95	41.4	24.01
7	Chronic EtOH Treated	Adult	3	35.29	52.94	18	18	34.78	26	36.4	11	17	19	11	27	11.33	20.51	31.99	22.63
10	Chronic EtOH Treated	Adult	3	46.15	56.25	14	17	36.84	41	40.9	7	10	9	16	22	0.71	26.43	37.07	27
53	Chronic EtOH Treated	Adult	3	87.5	71.43	9	8	29.41	20	29.41	10	9	20	12	22	19.3	13.95	41.4	24.01
61	Chronic EtOH Treated	Adult	3	55.55	80	10	11	26.83	44	27.3	11	12	15	12	16	6.67	5.98	32.46	12.38
75	Chronic EtOH Treated	Adult	3	77.78	50	10	15	28.57	38	40.9	10	30	31	13	17	6.63	9.34	34	18.39
81	Chronic EtOH Treated	Adult	3	50	66.66	7	10	32.43	40	31.8	18	15	20	15	24	4.48	6.87	18.78	30.52
89	Chronic EtOH Treated	Adult	3	42.86	48	21	26	14.63	43	9.09	10	6	29	11	25	10.93	15.13	40.85	26.11
56	Water Treatment Control	Adult	3	66.66	57.14	7	8	45.83	27	50	10	6	18	6	22	16.95	9.12	34.09	21.22
60	Water Treatment Control	Adult	3	83.33	44.44	13	10	40.91	25	40.9	8	6	14	8	18	11.51	15.58	33.64	28.65
62	Water Treatment Control	Adult	3	54.54	57.89	12	20	50	27	50	6	11	9	8	18		21.03	77.78	25.48
68	Water Treatment Control	Adult	3	80	47.06	11	18	35.71	17	35.71	9	9	22	13	15		18.51	40.25	32.65
74	Water Treatment Control	Adult	3	55.55	50	9	18	36.84	22	36.84	6	9	21	6	15	8.61	13.21	23.25	16.06
76	Water Treatment Control	Adult	3	54.54	66.66	12	7	34.29	38	27.3	12	9	10	11	9	19	8.93	42.89	26.27
80	Water Treatment Control	Adult	3	71.43	66.66	8	7	30	13	30	10	8	18	6	8	18.01	3.81	25.88	20.95
85	Water Treatment Control	Adult	3	58.33	78.57	13	14	32.14	31	36.36	15	12	8	7	6		16.41	36.68	30.73
