# Supplementary material for: Chronic Drinking During Adolescence Predisposes the Adult Rat for Continued Heavy Drinking: Neurotrophin and Behavioral Adaptation after Long-Term, Continuous Ethanol Exposure
Source: PLoS One. 2016 Mar 1;11(3):e0149987. doi: 10.1371/journal.pone.0149987 (PMC4773001; doi:10.1371/journal.pone.0149987)
Supplement: S2 Data — Estimated daily EtOH consumption (g/kg) as a function of ethanol concentration (6%, 9%, 12%, 20%) for 28 weeks for rats that started drinking at either postnatal day 35 (see Age at Onset; Adolescent) or postnatal days 73–75 (see Age at Onset; Adult). Brains were collected at 3 time points (Timepoint): 1 = During ethanol consumption; 2 = 48-hours after ethanol removal; 3 = 6 to 8 weeks post ethanol exposure and after behavioral testing. (PDF) [file pone.0149987.s002.pdf]

Supplementary Data Set 2

Rat ID	Treatment	Age at Onset	Timepoint	6%	9%	12%	20%	20%	20%	20%	20%	20%	20%	20%	20%	20%	20%	20%	20%	20%	20%	20%	20%	20%
	**Week**						**1**	**2**	**3**	**4**	**5**	**6**	**7**	**8**	**9**	**10**	**11**	**12**	**13**	**14**	**15**	**16**	**17**	**18**
11	Chronic EtOH Treated	Adolescent	1	8.861	9.807	13.52	15.61	21.01	12.09	18.65	16.73	14.4	26.56	20.82	16.28	10.12	10.33	10.76	8.991	10.43	9.142	10.45	11.88	15.61
13	Chronic EtOH Treated	Adolescent	2	8.782	9.551	13.25	15	19.77	11.83	16.87	16.16	13	23.96	18.98	14.94	9.099	9.156	9.488	7.966	9.446	8.515	9.836	11.44	15.15
15	Chronic EtOH Treated	Adolescent	3	11.52	17.02	24.65	15.33	17.4	15.41	23.64	26.02	18.11	14.93	16.65	11.64	9.667	10.48	11.6	10.23	12.89	11.03	13.53	11	18.51
17	Chronic EtOH Treated	Adolescent	1	11.74	18.12	26.58	16.58	19.8	17.46	27.23	29.12	19.72	16.11	18.54	13.04	10.62	11.79	13.09	11.43	14.32	12.28	15.05	11.9	20.73
19	Chronic EtOH Treated	Adolescent	2	10.62	13.2	15.87	18.6	14.5	16.11	14.09	15.12	11.01	13.83	17.16	10.78	10.37	9.48	9.164	9.638	9.178	10.26	10.34	11.24	11.51
21	Chronic EtOH Treated	Adolescent	2	7.537	8.501	12.79	14.12	11.56	12.7	13.99	14.23	11.48	16.08	15.96	12.75	9.395	9.993	9.807	10.69	9.312	9.638	10.36	22.43	11.82
23	Chronic EtOH Treated	Adolescent	3	7.242	9.704	11.86	12.08	13.01	11.39	12	12.12	9.705	13.94	13.76	11.11	8.205	8.817	8.497	9.231	8.178	8.761	9.318	20.13	10.64
25	Chronic EtOH Treated	Adolescent	1	8.299	8.69	13.54	14.46	18.38	12.86	13.73	13.9	11.48	16.35	15.37	12.55	9.299	9.892	9.663	10.32	9.131	9.684	10.26	22.13	11.87
27	Chronic EtOH Treated	Adolescent	2	10.2	25.87	22.59	12.99	20.38	13.1	18.35	19.46	14.21	19.24	23.48	22.72	11.93	10.2	9.26	9.325	10.41	9.796	10.72	11.78	12.3
29	Chronic EtOH Treated	Adolescent	3	11.21	27.93	23.83	13.95	9.239	15.44	20.16	19.65	12.61	16.95	20.91	20.85	11.1	9.451	8.565	8.803	10.04	9.456	10.56	11.74	11.77
31	Chronic EtOH Treated	Adolescent	1	8.011	8.902	15.14	19.29	17.69	18.74	20.39	22.36	19.39	16.74	19.15	16.47	11.99	10.04	8.594	8.464	10.95	9.684	13.19	10.12	14.72
33	Chronic EtOH Treated	Adolescent	2	8.322	8.554	14.93	19.29	17.92	16.83	17.86	18.2	16.26	14.29	16.27	14.2	10.44	8.638	7.341	7.071	9.13	8.096	11.15	8.631	12.52
35	Chronic EtOH Treated	Adolescent	3	8.011	26.36	14.72	19.29	18.32	17.49	18.69	20.7	18	14.9	17.8	15.28	11.14	9.247	7.85	7.756	9.871	8.612	11.94	9.076	13.01
37	Chronic EtOH Treated	Adolescent	3	10.67	12.89	15.48	16.75	12.86	14.53	15.39	13.75	10.32	12.83	15.55	9.784	9.291	8.431	8.247	8.345	8.411	9.448	9.405	10.27	10.18
39	Chronic EtOH Treated	Adolescent	1	10.09	10.29	10.78	23.23	17.1	18.31	15.56	17.96	15.88	15.47	17.43	15.73	14.47	14.63	11.95	10.7	10.75	12.62	18.1	15.63	14.5
41	Chronic EtOH Treated	Adolescent	2	10.43	11.13	11.47	24.33	16.8	19.72	16.63	19.95	17.38	16.13	18.65	16.86	15.57	15.76	12.95	11.85	12.1	13.84	19.94	16.81	16.16
43	Chronic EtOH Treated	Adolescent	3	9.917	8.867	17.42	16.97	12.33	12.71	12.51	11.84	10.75	10.09	11.53	10.66	9.238	9.428	8.993	8.912	8.949	8.985	10.26	10.61	11.33
44	Chronic EtOH Treated	Adolescent	3	7.095	11.59	12.69	12.84	17.86	15.87	20.77	11.19	14.16	12.07	10.88	13.87	10.06	9.687	10.16	10.25	9.578	10.13	13.4	13.59	11.9
45	Chronic EtOH Treated	Adolescent	1	5.353	13.33	10.72	14.8	18.04	13.73	13.05	12.01	11.19	13.01	11.15	7.681	9.68	9.364	10.24	9.287	9.379	8.943	9.602	9.875	12.39
46	Chronic EtOH Treated	Adolescent	2	5.261	13.38	10.69	15.1	19.09	14.83	13.49	12.13	11.9	13.63	12.16	8.488	10.42	10.17	10.94	9.906	9.926	9.398	10.05	10.35	12.98
48	Chronic EtOH Treated	Adolescent	2	6.317	9.498	10.92	11.44	13.14	15.54	17.26	15.17	19.41	18.55	14.29	10.18	10.34	13.81	11.43	14.69	14.79	18.36	13.17	10.36	11.03
50	Chronic EtOH Treated	Adolescent	3	4.721	9.186	9.147	9.866	9.834	11.41	11.07	7.817	10.37	10.09	9.152	8.499	8.429	9.075	8.183	6.693	8.129	8.879	18.5	10.03	9.668
52	Chronic EtOH Treated	Adolescent	1	6.164	9.427	10.7	11.36	13.82	16.34	17.44	14.24	18.46	17.84	14.35	10.32	10.43	13.75	11.73	15.45	15.16	19.36	13.75	10.76	11.42
91	Chronic EtOH Treated	Adolescent	1	6.381	12.16	11.83	12.55	14.01	14.11	14.67	11.14	13.95	12.87	11.59	10.38	9.938	10.79	9.875	8.107	9.961	11.08	23.18	12.73	12.56
1	Chronic EtOH Treated	Adult	2	4.045	6.396	6.963	4.873	10.32	8.308	8.286	8.258	8.499	8.384	7.81	8.112	8.763	10.55	10.82	11.29	11.62	11.33	7.875	8.894	10.91
3	Chronic EtOH Treated	Adult	3	3.842	6.368	7.057	4.941	10.71	8.835	8.846	8.832	8.941	8.853	8.238	8.581	8.974	10.97	10.98	11.33	11.8	11.54	7.964	8.961	10.83
4	Chronic EtOH Treated	Adult	1	5.846	9.976	10.43	10.89	12.76	10.02	9.808	11.24	11.9	8.952	9.426	10.91	9.334	10.05	10.4	12.74	10.41	12.08	12.58	14	8.619
5	Chronic EtOH Treated	Adult	2	6.518	12.73	11.5	5.197	12.78	12.13	9.849	8.477	9.562	14.55	12.47	13.03	10.95	8.512	9.499	9.167	7.914	11.87	7.691	8.002	8.499
6	Chronic EtOH Treated	Adult	1	5.824	11.98	10.99	4.9	12.12	11.04	9.029	7.418	8.228	12.91	11.33	11.81	10	7.609	8.381	8.167	7.038	10.84	6.861	7.074	7.35
7	Chronic EtOH Treated	Adult	3	5.726	13.84	8.874	5.884	11.49	8.608	9.157	10.99	11.74	10.8	9.534	11.62	10.33	9.793	11.01	9.667	9.667	9.635	10.84	11.02	12.15
9	Chronic EtOH Treated	Adult	1	5.781	13.53	8.754	5.695	11.13	8.91	9.279	11.14	11.63	11.14	9.576	11.72	10.55	9.834	11.33	9.904	9.745	9.991	10.92	11.56	12.59
10	Chronic EtOH Treated	Adult	3	5.297	12.93	8.769	7.477	11.7	7.869	7.945	8.183	10.38	11.2	11.42	12.32	9.661	11.03	8.814	9.691	7.789	7.807	8.867	8.263	9.138
53	Chronic EtOH Treated	Adult	3	5.301	10.13	8.341	9.704	11.13	12.99	8.697	8.645	8.685	6.405	8.539	8.402	8.439	7.507	8.685	14.59	11.05	8.762	6.513	8.002	9.759
59	Chronic EtOH Treated	Adult	1	5.484	6.277	8.441	18.15	7.806	12.17	12.19	13.01	11.71	7.232	11.98	16.56	8.441	7.644	9.456	9.234	8.122	10.73	8.955	8.079	8.805
61	Chronic EtOH Treated	Adult	3	5.828	7.832	10.48	9.574	8.593	11.83	12.83	11.98	13.21	11.02	19.04	11.29	12.02	13.98	12.85	11.59	11.03	9.288	9.961	10.71	11.53
65	Chronic EtOH Treated	Adult	1	4.536	6.452	6.961	7.237	9.717	9.893	10.11	14.52	13.72	8.382	16.93	9.853	9.634	10.14	8.431	8.63	7.759	8.803	9.723	10.01	10.4
69	Chronic EtOH Treated	Adult	2	4.795	6.198	6.727	8.722	9.576	8.534	9.464	8.722	7.724	9.082	8.403	7.397	7.897	8.146	8.875	8.864	8.035	8.288	5.953	5.914	6.322
71	Chronic EtOH Treated	Adult	1	4.483	5.811	6.501	8.428	9.576	8.534	9.683	9.042	8.046	9.328	8.864	7.897	8.494	8.8	9.332	9.277	8.548	8.751	6.202	6.467	6.871
73	Chronic EtOH Treated	Adult	2	3.916	5.165	5.638	7.492	8.236	7.524	8.537	8.013	7.123	8.346	7.734	6.902	7.436	7.64	8.299	8.293	7.413	7.167	5.679	5.588	5.938
75	Chronic EtOH Treated	Adult	3	5.304	8.069	7.944	11.55	9.449	11.03	12.42	10.16	12.46	11.63	10.81	10.57	8.981	10.55	10.04	9.234	11.29	9.433	10.26	13.1	12.32
77	Chronic EtOH Treated	Adult	2	5.355	8.107	7.835	11.13	9.11	10.55	12.65	10.21	12.68	11.68	10.72	10.52	8.981	10.55	9.915	9.234	11.24	9.966	10.5	13.6	12.59
79	Chronic EtOH Treated	Adult	2	6.439	9.548	9.262	14.5	10.83	13.18	11.2	8.273	12.33	10.46	10.94	9.151	11.36	11.29	11.45	10.97	10.13	9.665	9.71	9.581	9.04
81	Chronic EtOH Treated	Adult	3	6.504	9.928	9.756	15.49	11.61	14.04	11.72	8.79	13.08	11.48	12.34	10.24	12.74	12.65	12.98	12.18	11.29	10.93	11.09	10.75	9.967
83	Chronic EtOH Treated	Adult	1	6.375	9.687	9.391	14.84	10.63	13.13	11.4	8.162	12.17	10.55	10.94	9.227	11.75	11.61	11.84	11.33	10.46	9.933	10.09	10.07	8.835
87	Chronic EtOH Treated	Adult	2	8.546	7.07	8.288	7.654	13.18	10.98	9.385	8.42	12.16	10.66	8.871	11.61	12.39	11.13	12.88	8.85	11.57	10.95	12.43	10.82	11.06
89	Chronic EtOH Treated	Adult	3	5.298	7.242	7.364	5.767	17.85	8.783	9.457	7.979	9.843	9.601	8.601	9.167	12.83	11.06	21.77	10.8	11.24	13.51	9.766	10.6	11.33
90	Chronic EtOH Treated	Adult	2	5.102	6.921	7.036	5.41	16.43	8.156	8.511	7.089	8.798	8.487	7.657	8.363	11.76	10.35	20.34	10.09	10.57	12.92	9.234	9.81	10.46

**20%**	**20%**	**20%**	**20%**	**20%**	**20%**	**20%**	**20%**	**20%**	**20%**
**19**	**20**	**21**	**22**	**23**	**24**	**25**	**26**	**27**	**28**
12	11.91	12.79	9.598	12.24	12.8	13.81	12.43	11.93	8.774
11.88	11.67	12.33	9.38	12.2	12.92	13.72	12.47	11.81	8.746
8.91	15.16	11.4	10.94	8.726	7.896	10.44	10.76	8.324	7.343
9.729	16.45	12.27	11.88	9.301	8.432	11.12	11.52	8.98	8.124
13.29	9.272	11.24	10.48	11.69	10.87	11.24	10.26	7.946	8.454
10.15	7.797	9.875	18.38	9.413	6.775	10.21	13.22	8.167	10.94
9.345	6.973	8.955	16.76	8.732	6.213	9.449	12.33	7.554	10.14
10.37	7.693	9.831	18.22	9.413	6.714	10.3	13	8.063	10.85
11.93	8.251	12.74	15.46	10.47	7.538	10.59	12.61	9.84	10.74
11.97	7.751	11.68	14.61	9.933	6.741	9.673	11.91	9.007	10.02
9.148	9.898	10.59	13.68	9.315	9.66	8.94	17.42	12.7	19.89
7.866	8.566	8.881	11.54	7.999	8.351	7.659	15.02	10.88	16.85
8.08	8.873	9.122	11.67	8.09	8.443	7.77	15.24	11.07	17.46
11.76	7.952	9.77	9.13	10.45	9.411	9.958	9.021	7.266	7.573
12.92	16.84	15.62	14.62	9.698	6.725	10.74	11.8	8.096	10.13
13.96	18.51	17.06	16.12	10.86	7.013	11.29	12.41	8.548	10.89
10.19	8.523	8.357	8.101	11.08	11.24	10.34	9.399	8.494	9.729
14.66	12.52	9.601	10.61	11.53	6.783	17.25	14.7	9.411	10.56
14.77	13.78	10.81	10.38	15.82	9.073	9.429	9.256	9.214	8.703
15.06	14.29	11.03	10.65	16.51	9.475	9.642	9.465	9.454	8.87
12.59	10.05	11.49	8.564	10.03	9.219	6.377	8.427	10.35	8.306
6.921	9.321	8.294	8.824	7.867	8.654	7.726	8.692	7.311	8.794
13.41	10.57	12.22	9.045	10.54	9.993	6.846	9.061	11.24	8.956
8.828	11.52	10.47	11	10.06	11.19	9.996	11.2	9.374	11.33
10.26	9.405	9.045	9.292	7.892	8.405	14.09	17.03	10.53	12.13
10.15	9.205	8.76	8.944	7.757	8.261	13.65	16.51	10.46	11.67
7.935	8.521	8.136	8.242	7.487	9.515	7.524	13.02	8.184	9.796
8.292	7.859	9.147	10.62	8.228	9.338	7.449	7.985	9.037	10.97
7.255	6.883	8.016	9.491	7.221	8.268	6.477	6.966	7.95	9.656
7.362	10.9	9.13	9.26	8.443	7.759	7.649	7.579	8.271	9.433
7.661	11.33	9.56	9.723	8.802	8.024	8.068	7.926	8.711	9.798
7.079	9.372	8.211	8.991	10.32	8.306	8.884	10.68	10.26	11.52
11.1	12.38	8.788	19.01	14.54	8.171	6.205	8.029	7.347	7.494
8.287	6.532	8.061	9.418	8.966	8.435	8.007	7.399	8	7.459
11.69	8.961	10.2	10.41	9.893	10.61	7.493	11.75	9.68	8.577
12.92	8.595	9.463	9.544	8.782	10.28	8.251	11.84	10.12	9.556
5.852	5.912	6.49	5.643	6.906	4.332	5.414	5.705	6.621	6.238
6.311	6.471	7.115	6.013	7.403	4.825	5.988	6.383	7.085	6.701
5.574	5.616	6.183	5.277	6.682	4.129	5.112	5.352	6.503	5.815
11.54	12.55	12.03	10.68	9.499	7.178	9.332	9.418	8.07	8.328
11.67	12.79	12.21	11.03	9.781	7.441	8.983	10.19	8.485	8.719
12.19	9.517	10.24	9.845	10.06	9.835	9.802	10.2	8.848	8.48
13.88	10.67	11.47	11.08	11.29	11.15	11.23	11.49	10.05	9.871
13.26	10	10.88	10.19	10.48	10.35	10.27	10.81	9.412	12.06
10.7	9.886	9.508	9.973	9.234	7.84	8.421	10.38	11.16	11.46
8.787	9.526	10.12	8.36	8.844	9.23	9.915	12.23	11.29	11.79
8.222	8.791	9.36	7.574	8.204	8.558	9.224	11.66	10.68	8.539
