# Supplementary figures and images for: Chronic Drinking During Adolescence Predisposes the Adult Rat for Continued Heavy Drinking: Neurotrophin and Behavioral Adaptation after Long-Term, Continuous Ethanol Exposure
Source: PLoS One. 2016 Mar 1;11(3):e0149987. doi: 10.1371/journal.pone.0149987 (PMC4773001; doi:10.1371/journal.pone.0149987)

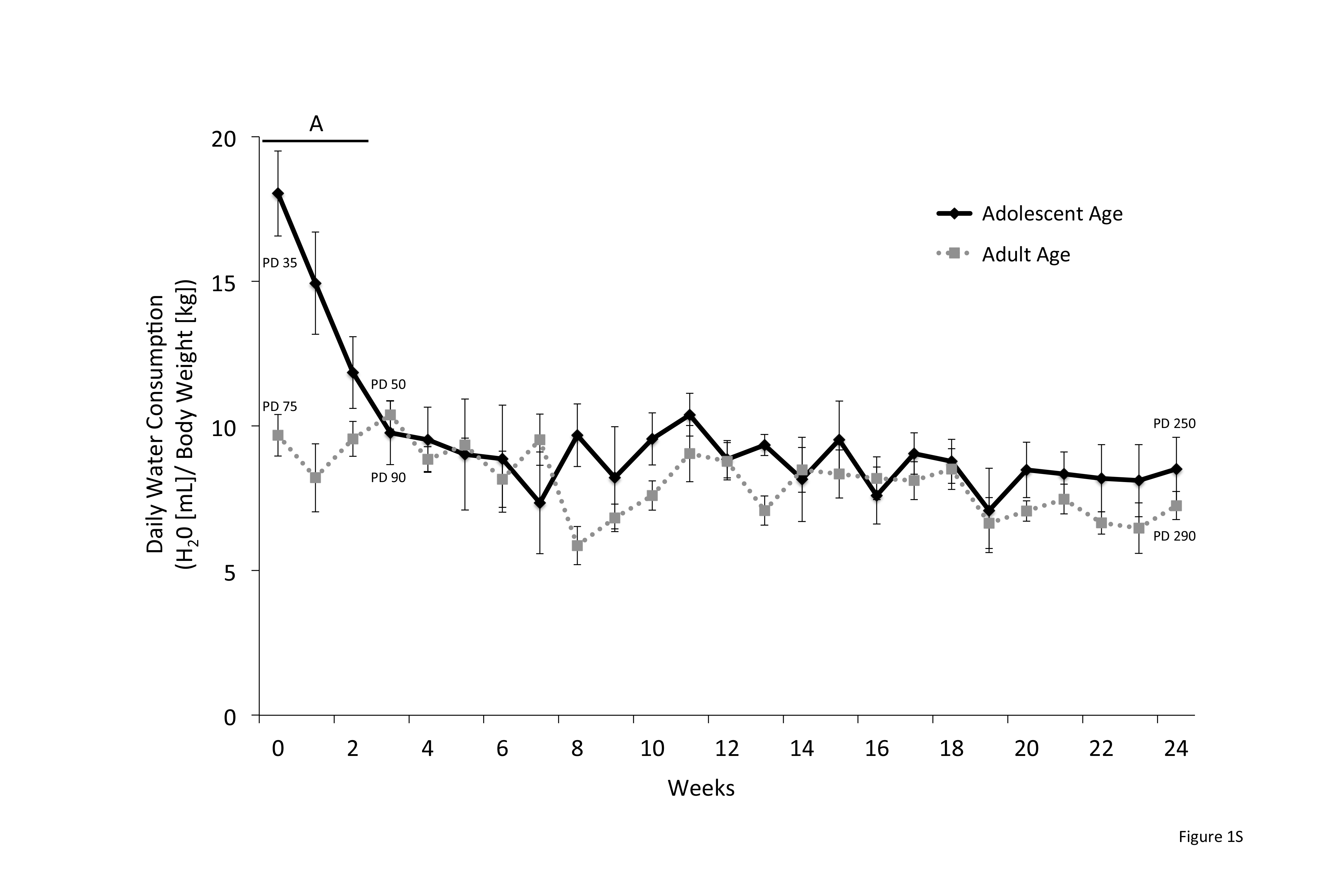

Supplement: S1 Fig — A difference in water consumption was observed between adult and adolescent age- matched animals on weeks 1–4 [A, p <0.05], where adolescent animals consumed significantly more liquid compared to adult age- matched rats. No further age dependent differences were found on consumption levels after week 4. (TIF) [file pone.0149987.s003.tif]
